# Genome-wide identification and expression profiles analysis of the authentic response regulator gene family in licorice

**DOI:** 10.3389/fpls.2023.1309802

**Published:** 2024-01-10

**Authors:** Yanping Shi, Guohua Ding, Haitao Shen, Zihan Li, Hongbin Li, Guanghui Xiao

**Affiliations:** ^1^ College of Life Sciences, Key Laboratory of Xinjiang Phytomedicine Resource and Utilization of Ministry of Education, Shihezi University, Shihezi, China; ^2^ College of Life Sciences, Shaanxi Normal University, Xi’an, China; ^3^ Geosystems Research Institute, Mississippi State University, Starkville, MS, United States

**Keywords:** ARR gene family, licorice, evolutionary analyses, expression patterns, stress responses, hormone

## Abstract

**Introduction:**

As one of the traditional Chinese medicinal herbs that were most generally used, licorice attracts lots of interest due to its therapeutic potential. Authentic response regulators (ARRs) are key factors in cytokinin signal transduction and crucial for plant growth and stress response processes. Nevertheless, the characteristics and functions of the licorice ARR genes are still unknown.

**Results:**

In present study, a systematic genome-wide identification and expression analysis of the licorice ARR gene family were conducted and 51 ARR members were identified. Collinearity analysis revealed the significant roles of segmental duplications in the expansion of licorice ARR genes. The cis-acting elements associated with development, stress and phytohormone responses were identified, implying their pivotal roles in diverse regulatory processes. RNA-seq and qRT-PCR results suggested that A-type, but not B-type ARRs were induced by zeatin. Additionally, ARRs participated in diverse abiotic stresses and phytohormones responses. Yeast one-hybrid assay demonstrated that GuARR1, GuARR2, GuARR11, GuARR12, GuARR10-1, GuARR10-2 and GuARR14 were able to bind to the promoter of GuARR8-3, and GuARR1, GuARR12 bound to the GuARR8-1 promoter. GuARR1, GuARR2, GuARR11 and GuARR10-2 bound to the GuARR6-2 promoter as well as GuARR12 and GuARR10-2 bound to the GuARR6-1 promoter.

**Discussion:**

Collectively, these findings provide a basis for future ARR genes function investigations, shedding light on the potential medicinal properties and agricultural applications of licorice.

## 1 Introduction

Cytokinins, initially discovered for their ability in stimulating cell division and modulating root and shoot initiation in culture and have been reported to participate in heterogeneous important biological processes afterward, are essential plant hormones for plant growth, development, and various stress responses (Miller et al., 1955; Miller et al., 1956; Hwang et al., 2002; Heyl and Schmülling, 2003; Kakimoto, 2003; Mizuno, 2004; Ferreira and Kieber, 2005; Aloni et al., 2006). A multi-step phosphorelay system were involved in the cytokinin signal transduction pathway, which comprises three main components: receptor protein histidine kinases (HKs), phosphoric acid group transfer proteins histidine-containing phosphotransfer proteins (HPs), and response regulator (RRs) (Hwang and Sheen, 2001; Müller and Sheen, 2007). In *Arabidopsis*, serving as transmembrane cytokinin receptors, the HKs (AHK2, AHK3 and AHK4/CYTOKININ RESPONSE 1 (CRE1)/WOODEN LEG (WOL)) can be autophosphorylated on the histidine (His or H) residue and transmit cytokinin signal to HPs (AHP1~AHP5) (Hwang and Sheen, 2001; Inoue et al., 2001; Hutchison et al., 2006; Kim et al., 2012). The phosphorylation group is finally transmitted to the aspartate (Asp or D) residue of ARRs from AHPs in the nucleus, modulating transcription of the downstream genes directly or indirectly (To et al., 2004; Mason et al., 2005; Hill et al., 2013). And among them, the ARRs are central regulators (Jeon and Kim, 2013).

The ARR family members are generally subdivided into A- and B-type ARRs and they all possess a N-terminal receiver domain approximately 120 amino acids harbouring a phosphorylatable invariant Asp residue which are highly conserved (Imamura et al., 1999; Imamura et al., 2003; Schaller et al., 2007; To et al., 2007; Hwang et al., 2012). The A-type ARRs consists of 10 members, including ARR3, ARR4, ARR5, ARR6, ARR7, ARR8, ARR9, ARR15, ARR16, and ARR17, which include a short C-terminal extension apart from the N-terminal receiver domain (To et al., 2007). Acting mainly as the negative-feedback regulation factors in cytokinin signaling, the transcription expression of the A-type *ARR* genes can be quickly activated by cytokinin (Kieber and Schaller, 2018). The B-type ARRs comprises 11 members, including ARR1, ARR2, ARR10, ARR11, ARR12, ARR13, ARR14, ARR18, ARR19, ARR20, and ARR21, which hold the role of positive regulators in cytokinin signaling and transcriptional activators with potential nuclear localization controlling the transcription of amounts of targets, including A-type *ARRs* (Mason et al., 2005). Besides the receiver domain, each of them contains a long C-terminal region embodying a Myb-like DNA-binding domain, also named B-motifs or GARP domain (Hosoda et al., 2002). This domain is essential for their role in activating target genes and interacting with other regulators (Rubio et al., 2001; Safi et al., 2017). B-type ARRs are further divided into 3 subfamilies in *Arabidopsis*. The ARR members (ARR1/2/10/11/12/14/18) in subfamily 1 are associated with cytokinin signal transduction (Mason et al., 2005; Yokoyama et al., 2007). Except for ARR21 in subfamily 2, which can function similarly with subfamily 1 members in cytokinin signal transduction, another member in subfamilies 2 (ARR13) and two subfamily 3 members (ARR19/20) showed no pronounced effects on cytokinin sensitivity (Hill et al., 2013). Additionally, ARR23 was identified as a potential pseudogene B-type ARRs containing the phosphorylated Asp site but a partial B motif (Schaller et al., 2008). ARR22 and ARR24 pertain to C-type ARRs, containing short C-terminal extension and perfect receiver domains with conserved D residuals, whose sequences are distinct from A- and B-type ARRs and are not induced by cytokinin (Kiba et al., 2004; Schaller et al., 2008).

The functions of ARRs have been explored. ARR4 can modulate the circadian light input in *Arabidopsis* through interacting with PHYTOCHROME B (phyB), acting redundantly with its closest relative ARR3 (Salomé et al., 2006). ARR7 and ARR15 inhibit shoot development and cell multiplication, involving maintenance and regeneration of the meristem (Buechel et al., 2010). ARR4 and ARR8 contribute significantly to the formation of shoot callus (Osakabe et al., 2002). ARR3 and ARR10 participate in regulating the root growth via the multistep phosphorelay pathway, in which Ethylene Response 1 (ETR1) interacts with AHPs and then phosphorylated ARR10 (Zdarska et al., 2019). ARR6, ARR7, and ARR15 play redundant roles in negatively regulating the seed germination (Huang et al., 2017). ARR16 is invovled in the cytokinin signaling pathway, the light as well as jasmonic acid signaling pathways, promoting the growth of hypocotyl in blue light (Srivastava et al., 2019). ARR16/17, together with CLE9/10 and SPEECHLESS (SPCH), regulate the epidermal cell-type composition, producing appropriate numbers and proportions of epidermal cells and stomata for the maintenance of leaf performance in fluctuating environments (Vatén et al., 2018). The phenotype of the *arr1arr10arr12* triple mutant showed that ARR1/10/12 together regulate a wide array of downstream processes, including seed size, shoot cell division, light sensitivity and concentration of anthocyanin (Argyros et al., 2008). ARR10 directly targets *WUSCHEL* (*WUS*), resulting in enhanced shooting in tissue culture (Zubo et al., 2017). ARR1 interacts with the ARABIDOPSIS TRITHORAX-RELATED 2 (ATXR2) protein to bind to the promoter regions of *ARR5* and *ARR7*, preventing premature *WUS* activation and transiently repressing cytokinin signaling, thus ensuring the proper cell fate transition (Lee et al., 2021). Mutant phenotypes of rice B-type *RRs rr21/22/23* indicate that they participate in regulating sensitivity for cytokinin, growth of leaf and root, development of flower, fertilization, shape of trichome and inflorescence architecture (Worthen et al., 2019). Besides, ARRs are also reported to take part in plant stress response. The *arr1/10/12* triple mutant displays enhanced adaptation to drought, which is achieved through the induced anthocyanin biosynthesis, abscisic acid sensitivity, improved cell membrane integrity, and reduced stomatal aperture (Nguyen et al., 2016). ARR1 can mediate the cold-inducible A-type *ARRs* (*ARR5/6/7/15*) expression, acting as a cold response positive regulator (Jeon and Kim, 2013). ARR2 is able to bind the defense marker gene *PATHOGENESIS-RELATED 1* (*PR1*), provoking transcription of cytokinin-dependent defense gene, which promotes *Arabidopsis* resistance to *Pseudomonas syringae* and the binding was induced by the interaction between a salicylic acid response factor TGACG SEQUENCE-SPECIFIC BINDING PROTEIN (TGA) and ARR2 (Choi et al., 2010). Wheat *ARR* genes are related to dehydration stress, and different expression patterns were observed for the A- and B-type *ARRs* in response to cytokinin treatment (Gahlaut et al., 2014). *OsARR-B5/7/9/10/16/22/23* showed a response to alkaline salt stress (Rehman et al., 2022). Overexpression of soybean *RR2a* in *Arabidopsis* significantly improves alkali stress tolerance (Chen et al., 2018).

Recently, *ARR* family genes in a lot of species have been examined at length, such as in *Arabidopsis*, rice, tobacco, tomato, *Brassica napus* (*B. napus*), and peach (Hwang et al., 2012; Zeng et al., 2017; Wang et al., 2020; Lv et al., 2021; Jiang et al., 2022; Rehman et al., 2022). However, the ARRs in licorice have not been studied. Licorice, one kind of the traditional Chinese medicinal herbs used commonly with a history of at least 2,000 years, contains a variety of triterpenoids and flavonoids for medicinal purposes (Tang et al., 2015). Licorice has been reported to have abundant pharmacological properties, including anticancer (Zhang et al., 2021), antidiabetic (Yang et al., 2020), anti-inflammatory (Wang et al., 2019), immunoregulatory activities (Aipire et al., 2017), and antiviral (Sun et al., 2019) effects. In addition, it is widely used as a health food and natural sweetener. Due to its medicinal and industrial properties, licorice has become a valuable and attractive trade item, with the export quota bidding reaching 5,200 tons in China in 2022 (http://www.mofcom.gov.cn). In Chinese pharmacopoeia, *Glycyrrhiza uralensis* Fisch. (*G. uralensis*), *Glycyrrhiza glabra* L. (*G. glabra*), and *Glycyrrhiza inflata* Batalin (*G. inflata*) are defined as licorice (National Pharmacopoeia Committee, 2010). The long and complex evolutionary history, as well as the complex genome of licorice, make the analysis and study of gene function more challenging. Therefore, a detailed analysis of ARRs will help to understand how they participate in plant development and stress responses through cytokinin signaling. In this study, the genome-wide identification, expression analysis, and function prediction of the *ARR* gene family in *G. inflata*, *G. glabra* and *G. uralensis* were performed. A total of 51 *ARRs* were identified in the three licorice species, and their evolution and structure were analyzed. This study is valuable for further research on the function of *ARR* gene in licorice and for the cultivation of excellent licorice varieties.

## 2 Materials and methods

### 2.1 Identification and the physicochemical properties of licorice *ARRs*

The *G. inflata*, *G. uralensis* and *G. glabra* whole genome database were assembled by Wuhan Benagen Technology Co., Ltd. The conserved receiver domain (PF00072) and Myb-like DNA-binding domain (PF00249) Hidden Markov Model (HMM) files were downloaded from Pfam (http://pfam.xfam.org/) and used to characterize the ARRs using HMMER software (Potter et al., 2018; El-Gebali et al., 2019). To identify the ARRs in these species, 24 *Arabidopsis* ARR protein sequences obtained from TAIR database (https://www.arabidopsis.org/) were used as queries to search against the licorice protein databases via the BLASTP program with the e-value 1e-5. The results from both HMMER and BLASTP hits were compared to remove redundancies. Additionally, the NCBI-CDD web server (http://www.ncbi.nlm.nih.gov/Structure/cdd/wrpsb.cgi) and SMART database (http://smart.embl-heidelberg.de) were employed to confirm the licorice ARRs. To learn more about the physicochemical properties of ARRs, ExPASy (http://web.expasy.org/compute_pi/) was used to determine the grand average of hydropathicity (GRAVY), molecular weight, and theoretical isoelectric point (pI) of the identified ARRs (Gasteiger et al., 2003). Furthermore, the subcellular localization of *ARRs* was predicted applying Cell-PLoc 2.0 (http://www.csbio.sjtu.edu.cn/bioinf/Cell-PLoc-2/).

### 2.2 Multiple sequence alignment and phylogenetic analysis

A multiple alignment of ARRs protein sequences from licorice and *Arabidopsis* was performed using ClustalX software with the default settings. Based on the alignment results, an unrooted phylogenetic tree was built through the Neighbor-Joining (NJ) method in MEGA7.0 following the parameters: p-distance, pairwise deletion and bootstrap values (1000 replicates) (Kumar et al., 2016). And the phylogenetic tree was visualized by Evolview v3.

### 2.3 Gene structure, conserved domain and the conserved motif analysis of the licorice *ARRs*

The conserved motifs of licorice ARR proteins in *G. glabra*, *G. inflata* and *G. uralensis* were analyzed through MEME (https://meme-suite.org/meme/) (Bailey et al., 2015). To gain more information about the gene structure characteristics, the GFF annotation information of *G. glabra*, *G. inflata* and *G. uralensis* genome was used to map the intron-exon distributions using TBtools (Chen et al., 2020). The licorice ARR protein sequences were submitted to the Pfam database for conserved domain analysis, and then visualized by TBtools.

### 2.4 Chromosomal location and duplication analysis of licorice *ARRs*

The chromosomal location information of licorice *ARRs* was extracted from the *G. glabra*, *G. uralensis* and *G. inflata* genome annotation files, and then visualized through MapChart. Multiple Collinearity Scan toolkit (MCScanX) was used to identify duplication events in each licorice genomes. The synteny of *ARRs* among three licorices were analyzed using MCScanX and visualized through the Dual Systeny Plotter function in TBtools. The KaKs_Calculator 2.0 was used to determine nonsynonymous (Ka) and synonymous (Ks) substitutions and Ka/Ks ratio between each gene pair (Wang et al., 2010).

### 2.5 Analysis of *cis*-acting elements in promoters

The 2 kb upstream sequences of *ARRs* were extracted by the Gtf/Gff3 Sequences Extractor function in TBtools and submitted to PlantCARE databases for the prediction of *cis*-acting regulatory elements (Lescot et al., 2002).

### 2.6 Plant materials and stress and hormone treatments

The *G. uralensis* seeds dormancy were broken by treating them with 98% concentrated H_2_SO_4_ for 50 minutes, followed by the wash with sterilized distilled water for three times. The treated seeds were then cultured in the mixture of soil and vermiculite (2:1, v:v) in the automatic climate chamber with the culture temperature of 28°C during the day and 25°C at night and a relative humidity of 50-55%. The photoperiod was 16 h of light and 8 h of darkness. The 60-day-old seedlings were transferred into Hoagland solution medium added 150 mM NaCl and 10% polyethylene glycol 6000 (PEG6000) for stress treatment. The roots of *G. uralensis* were collected after 0 h (the control group), 2 h, 6 h, and 12 h treatment. For hormone treatment, 60-day-old seedlings were treated with Hoagland solution medium containing 50 mM ABA, 100 µM methyl jasmonate (MeJA), 100 µM gibberellin (GA), and 100 µM auxin (IAA) for 0 h (the control group), 2 h, 6 h, and 12 h, respectively. In addition, 60-day-old seedlings were treated with Hoagland solution medium containing 0 (the control group), 15, 30, 50, 100 μmol/L zeatin for 6 h. Three biological replicates containting the roots of 15 seedlings in each group were pooled then quickly frozen in liquid nitrogen and stored at -80°C for further use.

### 2.7 RNA sequencing and quantitative real-time PCR analysis

Leaves and roots samples of *G. uralensis* treated with 0, 15, 30, 50, 100 μmol/L zeatin for 6 h were used to performed the RNA_seq, which were conducted by Shanghai Majorbio Bio-pharm Biotechnology Co., Ltd. (Shanghai, China) on the Illumina Novaseq 6000 platform (Illumina, San Diego, CA). After trimmed and cleaned by software SeqPrep (https://github.com/jstjohn/SeqPrep) and Sickle (https://github.com/najoshi/sickle), the cleaned data were obtained and then used for *de novo* assembly using software Trinity (http://trinityrnaseq.sourceforge.net/). After that, the clean data were compared with the downloaded *G. uralensis* genome through the TopHat2 (http://ccb.jhu.edu/software/tophat/index.shtml) and HISAT2 (http://ccb.jhu.edu/software/hisat2/index.shtml) softwares. The expressiones of *GuARRs* were normalized as Transcripts Per Million (TPM) values. Total RNA from each sample was extracted with the RNAprep Pure Plant kit (TIANGEN BIOTECH, Beijing, China) and first strand cDNAs were synthesized using the EasyScript One-step gDNA Removal and cDNA Synthesis SuperMix (Vazyme, Nanjing, China). The primers for qRT-PCR were designed through Primer Premier 5.0 and synthesized by TSINKE Biotech (Beijing, China), which were listed in **Supplementary Table S1**. *Guactin* (NCBI accession number: EU190972.1) was used as the internal control. The qRT-PCR analysis were performed using the 2×ChamQ SYBR qPCR Master Mix (Vazyme, Nanjing, China) on the Bio-RAD CFX96 Real-Time system (Hercules, CA, USA). The expression level was calculated using 2^−ΔΔCt^ method.

### 2.8 Yeast one hybrid assays

The 2,000 bp fragments from A-type licorice *ARRs* promoters were cloned individually into the pLaczi vector. The B-type licorice *ARRs* coding sequences were cloned into pJG-45 vector. Co-transformed into EGY48 yeast strain, the respective combinations were then selected on SD/-Trp-Ura dropout media. Positive transformants were transferred to the selection medium containing X-gal (5-bromo-4-chloro-3-indolyl-b-D-galactopyranoside) to develop a blue color.

## 3 Results

### 3.1 Identification and physicochemical properties of the *ARR* family genes in licorice

The licorice ARRs were initially identified by searching for conserved response regulator domains. Subsequently, a BLASTP search applying *Arabidopsis* ARRs sequences as the queries was performed to identify ARRs in the licorice protein database. The results from HMMER and BLASTP were combined to obtain putative licorice ARRs. After removing redundant sequences and sequences lacking complete domains, a total of 51 licorice ARRs were obtained, including 17 *G. glabra* ARRs (GgARRs), 17 *G. uralensis* ARRs (GuARRs), and 17 *G. inflata* ARRs (GiARRs). **Supplementary Table S2** presented their amino acid sequences. Among the identified ARRs, 7 GuARRs, 7 GgARRs, and 7 GiARRs contained both the response regulator domain and the Myb-like DNA-binding domain, classifying them as B-type ARRs. The amino acid length of these ARRs ranged from 124 to 700 aa. Their theoretical molecular weights ranged from 13.82 to 76.42 kDa, with an average of 42.53 kDa. Their theoretical isoelectric point (pI) ranged from 4.89 to 8.69 and the GRAVY from -0.916 to -0.042. Subcellular localization analysis predicted their nucleus locations (**Table 1**, **Supplementary Table S3**).

**Table 1:** Informations of ARR proteins in three licorice species.

Species	Gene name	Gene Id	Protein Length (aa)	Molecular weight (kDa)	Theoretical pI	GRAVY	Subcellular location
*G. uralensis*	*GuARR11*	Glycyrrhiza_uralensis_Fisch0107810	587	66.27	5.38	-0.537	Nucleus
	*GuARR10-1*	Glycyrrhiza_uralensis_Fisch0065040	641	70.63	6.07	-0.550	Nucleus
	*GuARR12*	Glycyrrhiza_uralensis_Fisch0240140	678	73.97	6.01	-0.488	Nucleus
	*GuARR10-2*	Glycyrrhiza_uralensis_Fisch0087060	669	73.21	6.02	-0.526	Nucleus
	*GuARR2*	Glycyrrhiza_uralensis_Fisch0196890	679	74.28	5.76	-0.557	Nucleus
	*GuARR1*	Glycyrrhiza_uralensis_Fisch0257130	685	74.51	5.83	-0.461	Nucleus
	*GuARR24-1*	Glycyrrhiza_uralensis_Fisch0071080	124	13.82	5.76	-0.042	Nucleus
	*GuARR24-2*	Glycyrrhiza_uralensis_Fisch0071060	131	14.58	5.68	-0.346	Nucleus
	*GuARR17*	Glycyrrhiza_uralensis_Fisch0093230	139	15.46	8.69	-0.241	Nucleus
	*GuARR14*	Glycyrrhiza_uralensis_Fisch0060960	668	72.88	6.42	-0.438	Nucleus
	*GuARR8-1*	Glycyrrhiza_uralensis_Fisch0067300	243	27.51	5.76	-0.916	Nucleus
	*GuARR8-4*	Glycyrrhiza_uralensis_Fisch0223710	179	20.4	5.60	-0.468	Nucleus
	*GuARR6-1*	Glycyrrhiza_uralensis_Fisch0104560	209	22.54	6.44	-0.178	Nucleus
	*GuARR8-2*	Glycyrrhiza_uralensis_Fisch0058800	269	30.87	6.00	-0.844	Nucleus
	*GuARR8-3*	Glycyrrhiza_uralensis_Fisch0094280	178	20.22	6.12	-0.475	Nucleus
	*GuARR6-2*	Glycyrrhiza_uralensis_Fisch0205420	212	23.53	6.20	-0.333	Nucleus
	*GuARR9*	Glycyrrhiza_uralensis_Fisch0061890	216	24.38	5.13	-0.392	Nucleus
*G. glabra*	*GgARR12*	GglaChr7G00201900	700	76.35	5.67	-0.484	Nucleus
	*GgARR10-1*	GglaChr2G00015200	643	70.83	6.32	-0.544	Nucleus
	*GgARR18*	GglaChr6G00259990	571	63.24	5.66	-0.361	Nucleus
	*GgARR10-2*	GglaChr3G00044390	669	73.4	6.15	-0.528	Nucleus
	*GgARR2*	GglaChr6G00264660	679	74.42	5.83	-0.557	Nucleus
	*GgARR1*	GglaChr7G00219490	685	74.49	5.90	-0.464	Nucleus
	*GgARR24-1*	GglaChr2G00009310	124	13.82	5.76	-0.042	Nucleus
	*GgARR24-2*	GglaChr2G00009320	131	14.57	5.68	-0.324	Nucleus
	*GgARR17*	GglaChr3G00050510	139	15.46	8.69	-0.241	Nucleus
	*GgARR3*	GglaChr5G00173910	229	24.56	5.24	-0.243	Nucleus
	*GgARR14*	GglaChr2G00019500	670	73.03	6.27	-0.429	Nucleus
	*GgARR8-2*	GglaChr6G00238080	177	20.17	5.60	-0.498	Nucleus
	*GgARR6-1*	GglaChr3G00061940	210	22.65	7.63	-0.196	Nucleus
	*GgARR8-1*	GglaChr2G00021650	269	30.87	6.00	-0.855	Nucleus
	*GgARR6-2*	GglaChr6G00256310	212	23.5	5.84	-0.317	Nucleus
	*GgARR9-1*	GglaChr2G00018600	216	24.38	5.13	-0.392	Nucleus
	*GgARR9-2*	GglaChr3G00072860	239	26.79	5.05	-0.618	Nucleus
*G. inflate*	*GiARR11*	GinfChr3G00136280	587	66.28	5.43	-0.547	Nucleus
	*GiARR18*	GinfChr6G00263800	571	63.23	5.58	-0.358	Nucleus
	*GiARR12*	GinfChr7G00226320	700	76.42	5.67	-0.490	Nucleus
	*GiARR10*	GinfChr3G00157450	669	73.47	6.15	-0.533	Nucleus
	*GiARR2*	GinfChr6G00268450	679	74.29	5.76	-0.549	Nucleus
	*GiARR1*	GinfChr7G00209560	685	74.5	5.83	-0.461	Nucleus
	*GiARR24-1*	GinfChr2G00030740	124	13.82	5.76	-0.042	Nucleus
	*GiARR24-2*	GinfChr2G00030730	131	14.6	5.68	-0.305	Nucleus
	*GiARR17*	GinfChr3G00151240	200	21.74	8.14	-0.114	Nucleus
	*GiARR8-1*	GinfChr2G00026960	243	27.36	5.75	-0.877	Nucleus
	*GiARR3*	GinfChr5G00177120	224	24.12	5.24	-0.227	Nucleus
	*GiARR14*	GinfChr2G00020490	670	73.01	6.33	-0.408	Nucleus
	*GiARR8-3*	GinfChr6G00241790	179	20.43	5.60	-0.454	Nucleus
	*GiARR6-1*	GinfChr3G00139830	210	22.66	7.63	-0.196	Nucleus
	*GiARR8-2*	GinfChr2G00018300	272	31.23	5.94	-0.868	Nucleus
	*GiARR6-2*	GinfChr6G00260050	212	23.48	5.83	-0.314	Nucleus
	*GiARR9*	GinfChr3G00129190	238	26.71	4.89	-0.630	Nucleus

### 3.2 Phylogenetic analysis of ARRs in licorice

**Figure 1:**
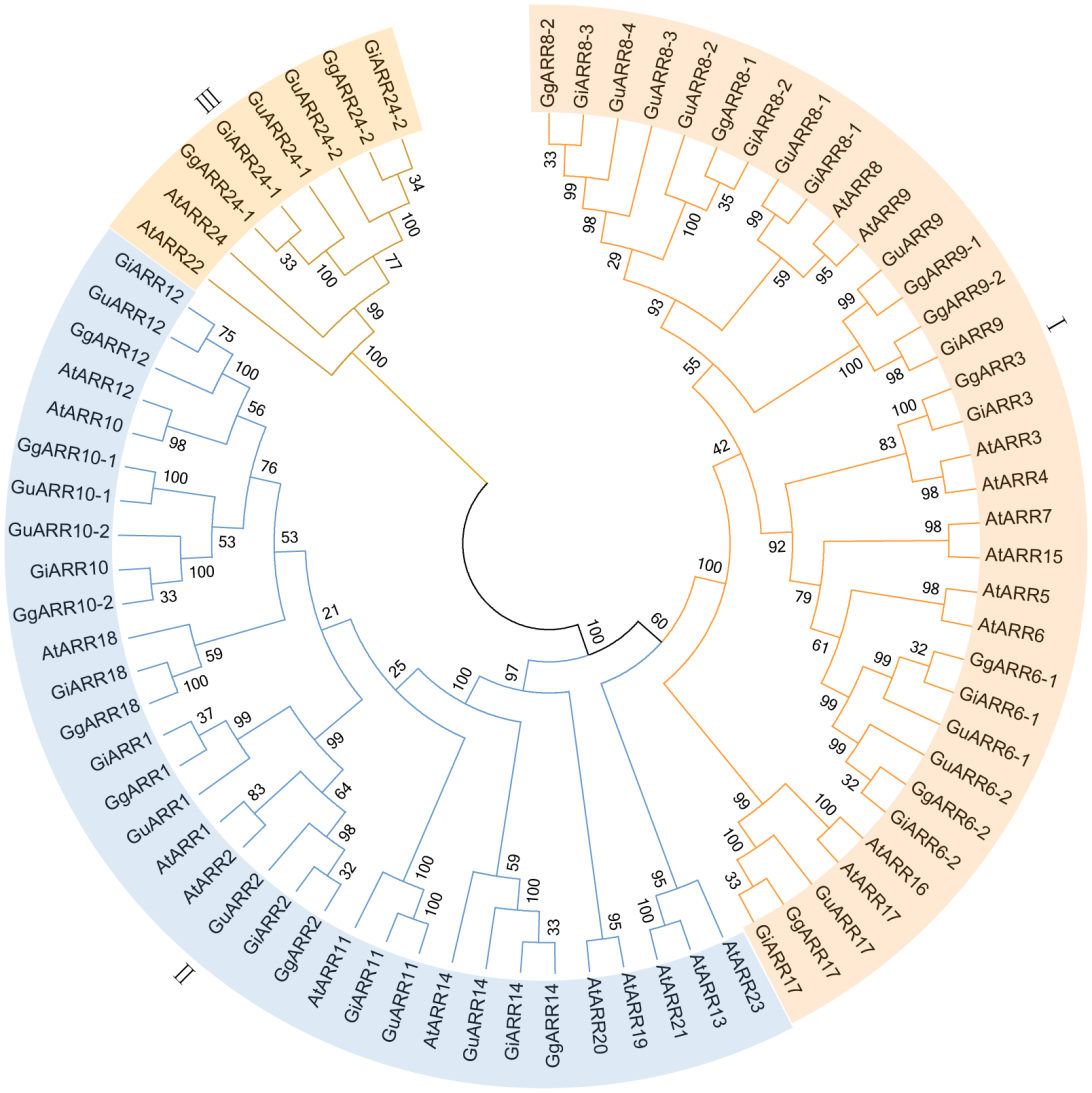
The neighbor-joining phylogenetic tree of ARRs from *G. inflata*, *G. uralensis*, *G. glabra* and *A. thaliana* using MEGA 7.0 based on their protein sequences. The three kinds of background colors indicated the three groups of the ARRs proteins.

To elucidate the evolutionary relationships of ARRs in *G. inflata*, *G. uralensis*, *G. glabra*, and *Arabidopsis*, we constructed an unrooted Neighbor-Joining (NJ) phylogenetic tree applying sequences of 24 AtARRs, 17 GuARRs, 17 GgARRs, and 17 GiARRs (**Figure 1**). The 75 ARRs were categorized into three groups, consistenting with the classification of A-, B-, and C-type ARRs in *Arabidopsis*. Group I comprised 34 A-type ARRs, including 10 AtARRs (AtARR3, AtARR4, AtARR5, AtARR6, AtARR7, AtARR8, AtARR9, AtARR15, AtARR16, AtARR17), 8 GgARRs (GgARR6-1, GgARR6-2, GgARR3, GgARR8-1, GgARR8-2, GgARR9-1, GgARR9-2, GgARR17), 8 GuARRs (GuARR6-1, GuARR6-2, GuARR17, GuARR8-1, GuARR8-2, GuARR8-3, GuARR8-4, GuARR9), and 8 GiARRs (GiARR6-1, GiARR6-2, GiARR3, GiARR17, GiARR8-1, GiARR8-2, GiARR8-3, GiARR9). Thirty-three B-type ARRs were included in groups II, which contained 12 AtARRs (AtARR19, AtARR14, AtARR20, AtARR18, AtARR11, AtARR2, AtARR1, AtARR10, AtARR12, AtARR13, AtARR21, AtARR23), 7 GgARRs (GgARR14, GgARR18, GgARR2, GgARR1, GgARR10-1, GgARR10-2, GgARR12), 7 GuARRs (GuARR14, GuARR11, GuARR2, GuARR1, GuARR10-1, GuARR10-2, GuARR12), and 7 GiARRs (GiARR14, GiARR18, GiARR11, GiARR2, GiARR1, GiARR12, GiARR10). Group III consisted of 2 AtARRs (AtARR22, AtARR24), 2 GgARRs (GgARR24-1, GgARR24-2), 2 GuARRs (GuARR24-1, GuARR24-2), and 2 GiARRs (GiARR24-1, GiARR24-2), which were regarded as C-type ARRs.

### 3.3 Sequence alignment of ARRs in licorice

**Figure 2:**
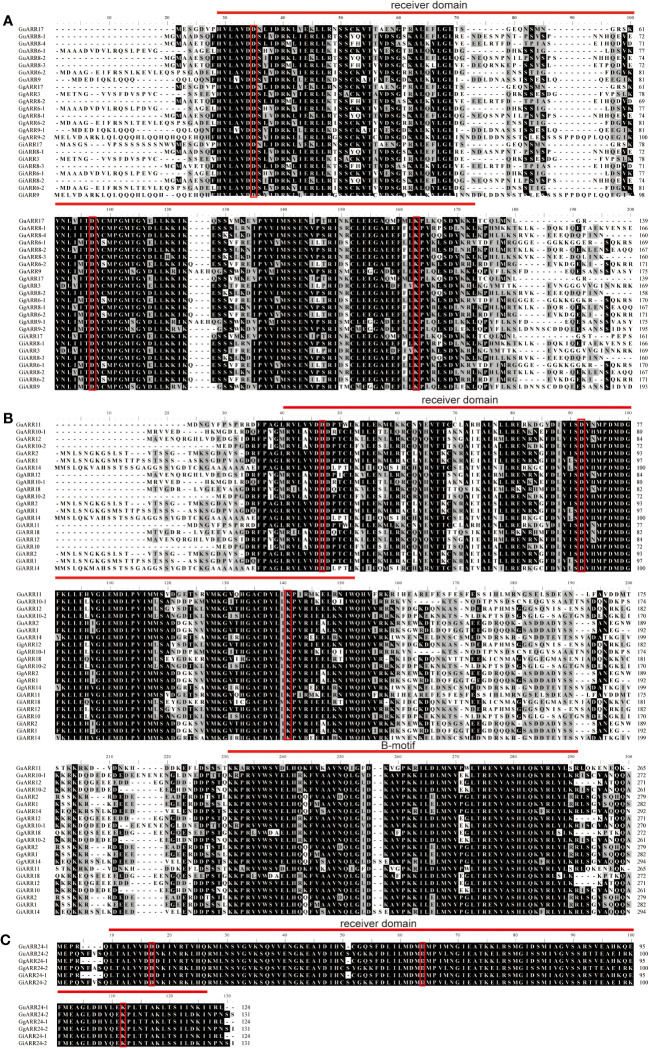
Multiple alignment of the three species of licorice ARRs proteins sequences. **(A)** Multiple alignment of A-type licorice ARRs proteins sequences. **(B)** Multiple alignment of B-type licorice ARRs proteins sequences. **(C)** Multiple alignment of C-type licorice ARRs proteins sequences. The conserved D-D-K residues were highlighted with red rectangles. The receiver domain and B-motif were highlighted with red lines.

To further investigate the features of the three types of licorices ARRs, the multiple sequence alignments were conducted. The results revealed that all licorice ARRs contained the typical conserved receiver domains containing approximately 120 amino acids, and three conserved phosphorylated amino acid residues existed in which, namely the centeral D1 site, the N-terminal D2 site, and the C-terminal lysine (K) site. A short insertion was exhibited in the A-type licorice ARRs receiver domains (**Figure 2**). These features of licorice ARRs sequences provided strong support for their classification based on the phylogenetic results.

### 3.4 Conserved motif, gene structure and structural domain analysis of *ARRs*

**Figure 3:**
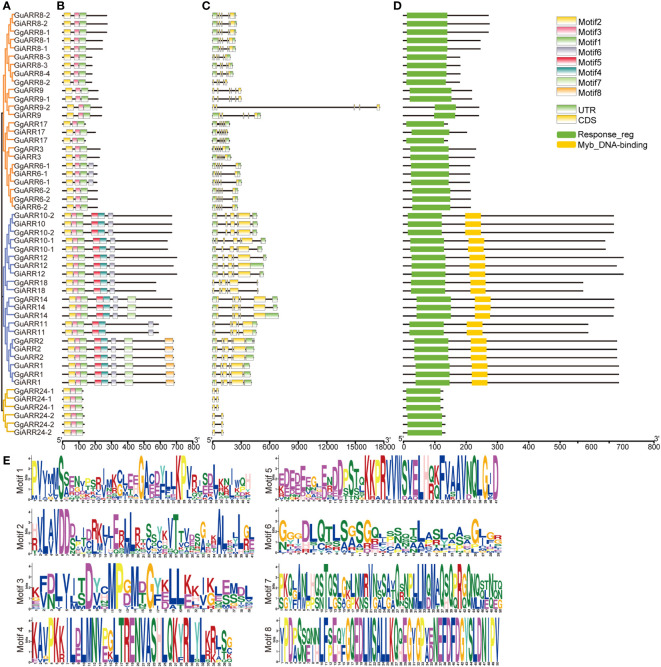
Phylogenetic tree, motifs composition, exon/intron structures and domains of ARRs in licorice. **(A)** Phylogenetic tree of licorice ARRs constructed with 1000 bootstrap replicates. **(B)** Conserved motifs of licorice ARRs proteins. A total of 8 motifs were shown. **(C)** Exon/intron structures of licorice *ARRs*. Black lines represented introns, yellow rectangles represented exons and green rectangles represented UTRs. **(D)** Conserved functional domains of licorice ARRs. Yellow rectangles represented Myb-like DNA-binding domains and green rectangles represented the conserved receiver domains. **(E)** Sequences logos of identified motifs.

The MEME analysis identified 8 motifs with a length of 29 to 50 amino acids (**Figure 3B**, **Table 2**). These motif sequences were further searched in Pfam for functional identification. Presented in all ARR proteins, motifs 1, 2, and 3 together formed the response regulator receiver domain (**Figures 3B, E**). Motifs 4 and 5 constituted the Myb-like DNA-binding domain, which were exclusively found in B-type ARRs (**Figures 3B, E**). The remaining motifs were designated as unknown motifs.

**Table 2:** Amino acid composition and function of different motifs.

Motif Number	Length (aa)	Best Possible Match	Function
Motif 1	42	PVVMMSSENVPSRIMKCLEEGACDYLLKPVRJSDLKNJWQH	Response regulator receiver domain
Motif 2	42	HVLAVDDDLIDRKLJERLLRTSSYKVTTVDSGIKALKLLGL	Response regulator receiver domain
Motif 3	29	KFBLVITDYCMPGMDGYKLLKKIKLEMDL	Response regulator receiver domain
Motif 4	42	KAVPKKILDLMNVPGLTRENVASHLQKYRLYLKRLSGVAQQ	Myb-like DNA-binding domain
Motif 5	42	EDEDEEGLENDDPSTQKKPRVVWSVELHQKFVAAVNQLGJD	Myb-like DNA-binding domain
Motif 6	29	GGGDLQTLSGSGQLSSQTLASLQAAGJGR	unmatched
Motif 7	51	PKQLANLHQSTQSLGNLNMRVNASAAQSNPLLMQMAQSQPRGQNQSENEN	unmatched
Motif 8	51	IPDPSNZNHLFPEHYGQEDLMSALLKQHEGIGPVDNEFDFDGYSLDNIPV	unmatched

A phylogenetic tree of the 51 licorice ARRs was built based on their full-length protein sequences by the NJ method to characterize their evolutionary relationships (**Figure 3A**). The results showed that these 51 ARRs were divided into three classes, which was consistent with the phylogenetic tree of *Arabidopsis* ARRs (**Figure 1**). Gene structures of the licorice *ARRs* were further investigated, and the numbers of their exon ranged from 2 to 6 (**Figure 3C**). Most of the A-type *ARRs* contained 5 exons, except for *GuARR9*, *GgARR9-1*, and *GiARR17*, which each contained 6 exons. Among the 21 licorice B-type *ARRs*, six members (*GuARR12*, *GgARR14*, *GuARR14*, *GiARR14*, *GuARR11*, and *GiARR11*) contained 5 exons, whereas the remaining B-type *ARRs* contained six exons. All six C-type *ARRs* contained two exons. In general, licorice *ARRs* within the same evolutionary tree branch exhibited similar structures on the exons number and length. To further understand the structure characteristics of the licorice ARR family protein sequences, the protein domains of the three types of licorice ARRs were analyzed using Pfam website and visualized with TBtools. The results displayed that all licorice ARR sequences contained the conserved receiver domain, but the Myb-like DNA-binding domain was present exclusively in B-type ARRs (**Figure 3D**).

### 3.5 Chromosome distribution and collinearity analysis of the *ARR* gene family in licorice

**Figure 4:**
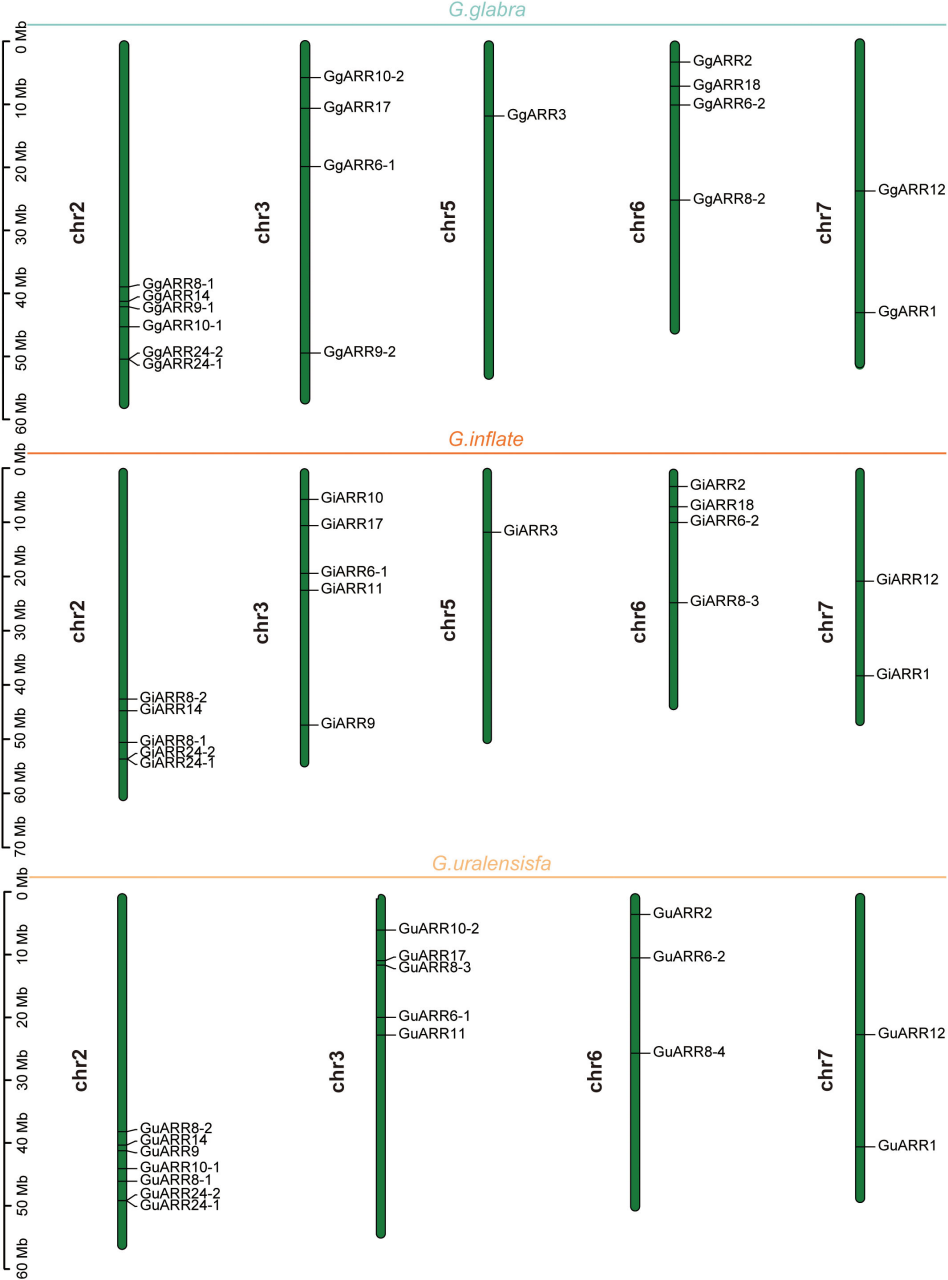
The chromosomal locations of the 51 *ARR* genes from *G. inflata, G. uralensis* and *G. glabra*. The vertical bar on the left side represented the length of chromosome. Mb, megabase.

**Figure 5:**
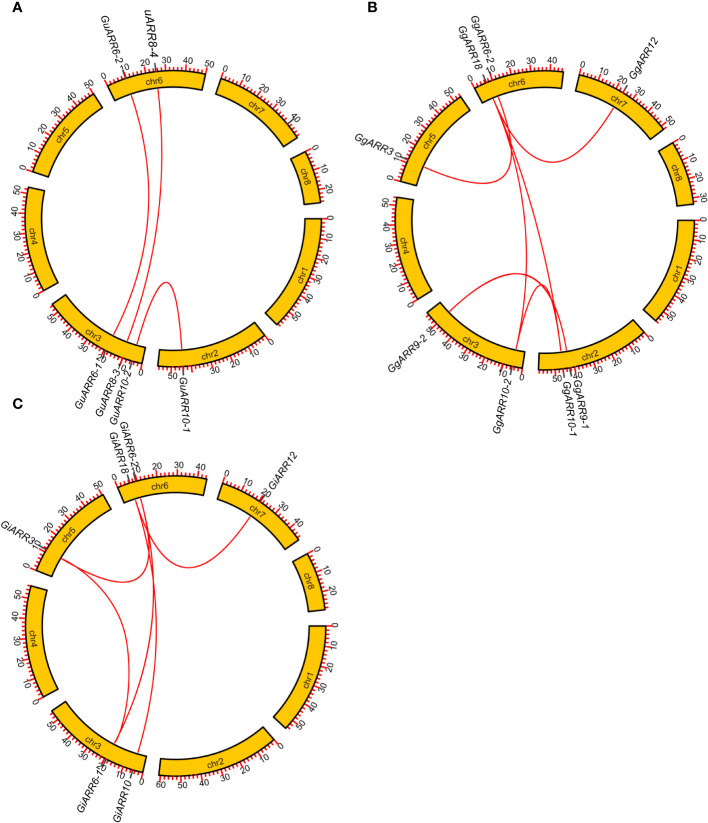
Gene duplication relationship among *ARRs* of *G*. *inflata*, *G. uralensis* and *G. glabra*. **(A)** Gene duplication relationship among *GuARRs*. **(B)** Gene duplication relationship among *GgARRs*. **(C)** Gene duplication relationship among *GiARRs*. The scale on the circle was in Megabases.

**Figure 6:**
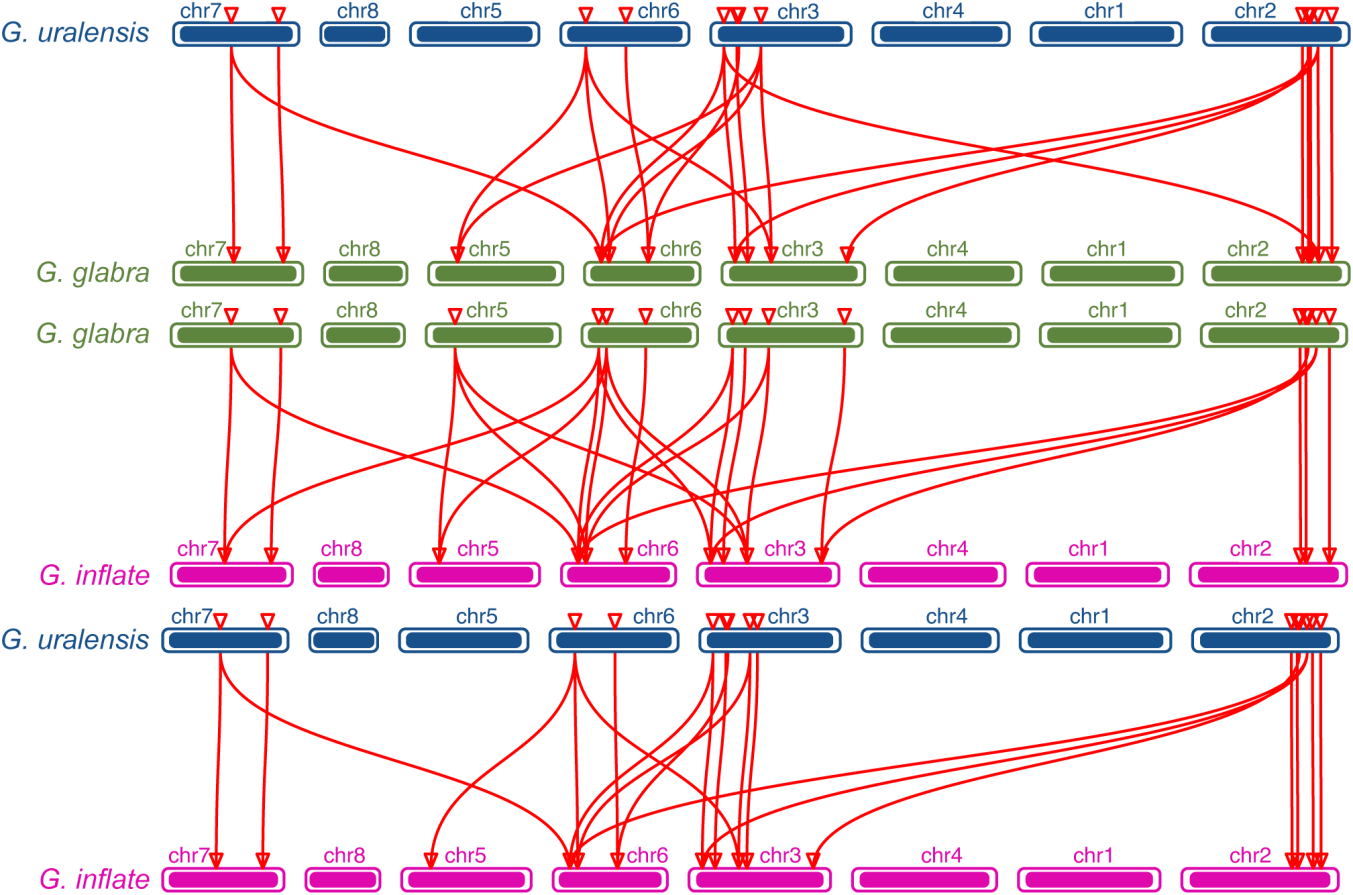
The collinearity relationship between *GuARRs* and *GgARRs*, *GgARRs* and *GiARRs*, *GuARRs* and *GiARRs*.

The chromosomal distributions of *GgARRs*, *GuARRs*, and *GiARRs* were visualized according to their genomic positions (**Figure 4**, **Supplementary Table S3**). In *G. uralensis*, 17 *GuARRs* were mapped on 4 chromosomes unevenly. Specifically, chr2 included the most number of *GuARRs*, with 7 members (*GuARR8-1/2*, *GuARR14*, *GuARR9*, *GuARR10-1*, *GuARR24-1/2*), and chr3 contained 5 *GuARRs* (*GuARR10-2*, *GuARR17*, *GuARR8-3*, *GuARR6-1*, *GuARR11*). In contrast, chr6 and chr7 had fewer *GuARR* genes, with three (*GuARR2*, *GuARR6-2*, *GuARR8-4*) and two (*GuARR1*, *GuARR12*) *GuARRs*, respectively. The *ARRs* distributions in *G. glabra* and *G. inflata* were similar, with genes located on chr2, 3, 5, 6, and 7. For the *GgARRs*, 6 members (*GgARR8-1*, *GgARR14*, *GgARR9-1*, *GgARR10-1* and *GgARR24-1/2*) were located on chr2. Chr3 and chr6 contained the same number of *ARRs*, with *GgARR10-2*, *GgARR17*, *GgARR6-1*, *GgARR9-2* located on chr3 and *GgARR2*, *GgARR6-2*, *GgARR8-2*, *GgARR18* on chr6. Chr7 comprised *GgARR1* and *GgARR12*. Only *GgARR3* was found on chr5. In the case of *GiARRs*, chr2 and chr3 had the same number of *GiARRs*. *GiARR8-1/2*, *GiARR14*, *GiARR24-1/2* were located on chr2, and *GiARR6-1*, *GiARR9/10/11/17* on chr3. *GiARR2*, *GiARR6-2*, *GiARR8-3*, *GiARR18* were located on chr6. As it was for *GgARRs*, chr7 of *G. inflata* contained *GiARR1* and *GiARR12*, and chr5 contained *GiARR3*. To understand the expansion mechanism of the *ARRs*, segmental and tandem duplications events within the licorice genomes were examined. The results showed that the licorice *ARR* gene family had no tandem duplication gene pairs. However, there were 5, 3, and 6 segmental duplication gene pairs in *G. inflata*, *G. uralensis*, and *G. glabra* genomes, respectively. Among these duplication relationships, *GiARR18* was paired with *GiARR12* and *GiARR10*, *GiARR6-2* was paired with *GiARR6-1* and *GiARR3*, *GgARR18* was paired with *GgARR12*, *GgARR10-1*, and *GgARR10-2*, respectively, while the others were one-to-one paired. *GgARR3* was paired with *GgARR6-2*, *GgARR9-1* was paired with *GgARR9-2*, *GgARR10-1* was paired with *GgARR10-2*. As for the *GuARRs*, *GuARR6-1* was paired with *GuARR6-2*, *GuARR8-3* was paired with *GuARR8-4*, *GuARR10-1* was paired with *GuARR10-2* (**Figure 5**). These results indicated that several licorice *ARR* genes were possibly generated through gene duplication, and the main driving force for licorice *ARR* gene evolution was segmental duplication. Additionally, comparative syntenic maps were constructed among the three licorices. The results revealed that 13 *GuARRs* had a syntenic relationship with 15 *GgARRs*, accompanying 23 orthologous pairs. Furthermore, there were 21 orthologous pairs between 15 *GuARRs* and 15 *GiARRs*, while 15 *GgARRs* and 13 *GiARRs* had 25 pairs of syntenic relationships (**Figure 6**; **Supplementary Table S4**). These findings suggested the significant roles of these genes in the *ARR* genes evolution. More than 88% of the 69 gene pairs had a ratio of nonsynonymous substitution rate (Ka) to synonymous substitution rate (Ks) below 1 (**Supplementary Table S4**), indicating purifying selection during evolution of these genes, and they have relatively conserved functions.

### 3.6 *Cis*-acting regulatory elements in the promoter of *ARR* genes

**Figure 7:**
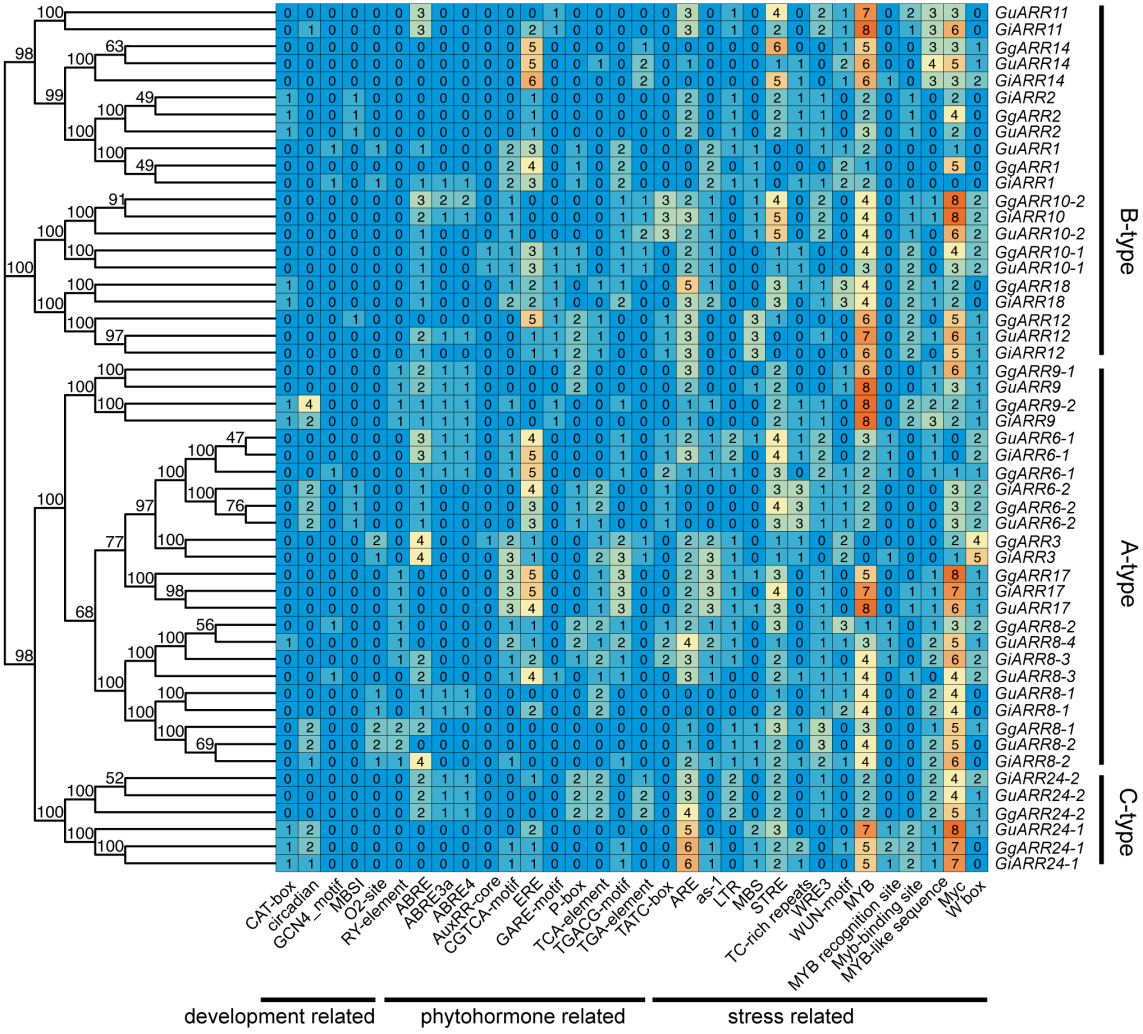
The numbers of growth and development, phytohormone and stress response related *cis*-acting elements were shown in licorice *ARRs* promoter regions. The phylogenetic tree of licorice ARRs was on the left and the classification of ARRs was on the right.

To grasp the potential function and regulatory mechanisms of licorice *ARR* genes, we analyzed their promoter sequences to identify potential *cis*-acting elements using the PlantCARE database. The analysis showed the existence of 6908 *cis*-acting elements, and 4904 among them were involved in promoter binding sites, including 285 AT~TATA box (4.13%), 1904 CAAT-box (27.56%), and 2715 TATA-box (39.30%) (**Supplementary Table S5**). Additionally, a quantity of light-responsive elements were also found in the licorice *ARR* promoters, including 160 Box 4, 84 G-Box, 45 GT1-motif, 43 GATA-motif, 34 TCT-motif, 31 TCCC-motif, 25 AE-box, 17 GA-motif, 15 I-box, 13 MRE, 11 ATCT-motif, 10 LAMP-element, 10 AT1-motif, 8 chs-CMA1a, 6 ATC-motif, 4 ACE and 4 chs-CMA2a, taking up 7.53% of the total *cis*-acting elements (**Supplementary Table S5**). Furthermore, there were 73 plant growth and development related *cis*-acting elements detected in the licorice *ARR* genes promoters, in which the circadian (involved in circadian control) was the most with 23, followed by 15 RY-element (seed-specific regulation related), 12 O2-site (zein metabolism regulation related), 11 CAT-box (meristem expression related), 7 MBSI (related to flavonoid biosynthetic gene regulation) and 5 GCN4 motif (involved in endosperm expression) (**Figure 7**). A total of 398 hormone-related *cis*-acting elements were existed, in which 18 TGA-element and 3 AuxRR-core were IAA responsive elements, 63 ABRE, 17 ABRE3a and 17 ABRE4 were ABA responsive elements, 32 TCA-element were salicylic acid responsive elements, 40 CGTCA-motif and 40 TGACG-motif were MeJA responsive elements, 12 GARE-motif, 29 P-box and 24 TATC-box were gibberellin responsive elements, and 103 ERE were ethylene response elements. Regarding the 1013 stress-related *cis*-acting elements, the analysis revealed the presence of 207 MYB, 207 Myc, 119 STRE, 107 ARE, 57 MYB-like sequence, 57 W box, 49 WRE3, 40 as-1, 37 WUN-motif, 36 Myb-binding site, 30 TC-rich repeats, 29 LTR, 26 MBS and 12 MYB recognition site. Overall, the distribution and composition of these diverse *cis*-acting elements may play a crucial role in shaping the expression of the *ARR* family genes in response to various stimuli, including light, hormones, abiotic stresses, and licorice plant development.

### 3.7 Expression pattern of *GuARRs*under abiotic stress and phytohormone treatments

**Figure 8:**
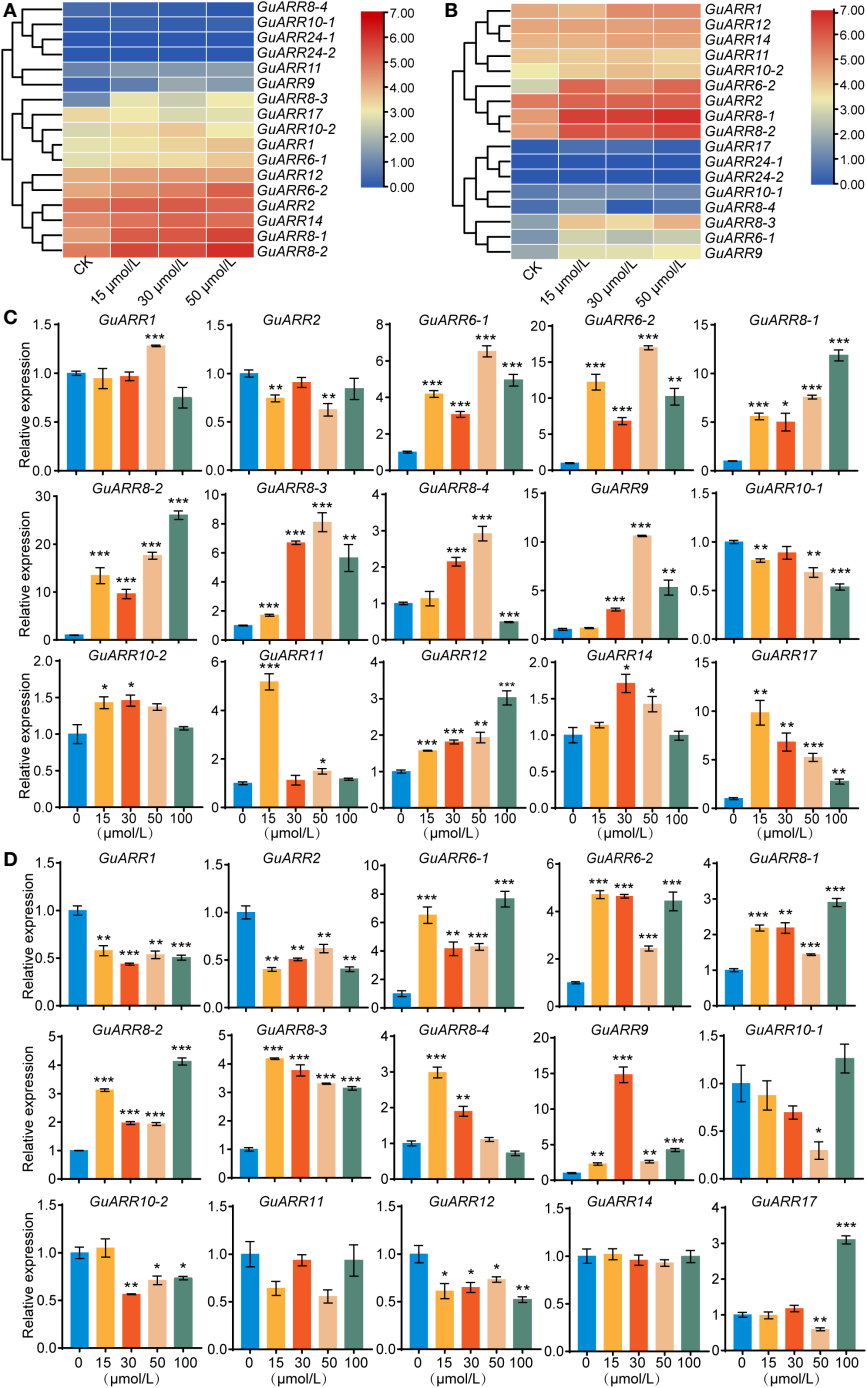
The heatmaps of the expression profiles of *GuARRs* under various concentrations of zeatin treatments. **(A)** Heatmap of *GuARRs* expression profiles in aboveground part. **(B)** Heatmap of *GuARRs* expression profiles in roots. The selected *GuARRs* expression levels in the licorice roots **(C)** and aboveground part **(D)** under various concentrations of zeatin treatments via qRT-PCR. Student’s t-test was used to assess significant differences. Significance levels: *, p < 0.05; **, p < 0.01,***, p < 0.001.

According to the RNA_seq data, the expression profiles of *GuARRs* under different concentrations of zeatin treatment were analyzed (**Figures 8A, B**). The expression results showed that *GuARR8-1/2/3*, *GuARR6-1/2*, *GuARR1* and *GuARR9* were increased in the aboveground part under zeatin treatment, whereas *GuARR8-4* was decreased (**Figure 8A**). In the root, the expressions of *GuARR8-1/2/3*, *GuARR6-1/2*, *GuARR9*, and *GuARR17* were significantly induced by zeatin treatment, and *GuARR8-4* was up-regulated under 15 μmol/L zeatin treatment (**Figure 8B**). And the C-type *GuARRs* had pretty low expression in both aboveground part and root as the TPM values were zero. To validate the RNA_seq data, qRT-PCR was conducted to explore the expressions of A-type and B-type *GuARRs* in the aboveground part and root under zeatin treatment (**Figures 8C, D**). QRT-PCR showed that most of the *GuARRs* showed similar expression patterns with the data from RNA_seq. In roots, A-type *GuARRs* (*GuARR6-1/2*, *GuARR8-1/2/3/4*, *GuARR9*, *GuARR17*) showed significant induction by zeatin treatment. *GuARR6-1* and *GuARR6-2* were induced with 6.53-folds and 16.98-folds after treatment of 50 μmol/L zeatin for 6 h, respectively. The expression of *GuARR8-1* and *GuARR8-2* were increased with 11.85-folds and 26.06-folds after treatment of 100 μmol/L zeatin, respectively. Expressions of *GuARR8-3*, *GuARR8-4* and *GuARR9* were up to 8.10-folds, 2.92-folds and 10.62-folds after treatment of 50 μmol/L zeatin, respectively. In aboveground part, the expression of *GuARR17* was up to 9.83-folds after 15 μmol/L zeatin treatment (**Figure 8C**). Expression of *GuARR9* was up to 14.82-folds after 30 μmol/L zeatin treatment. *GuARR17*, *GuARR6-1*, *GuARR8-1* and *GuARR8-2* were induced with 3.10-folds, 7.64-folds, 2.90-folds and 4.12-folds after treatment of 100 μmol/L zeatin, respectively. The expression of *GuARR6-2, GuARR8-3* and *GuARR8-4* were increased with 4.71-folds, 4.18-folds and 2.98-folds after treatment of 15 μmol/L zeatin, respectively (**Figure 8D**). These results were consistent with their cytokinin responsive characristic. However, some discrepancies were observed for B-type *GuARRs*, with *GuARR2*, *GuARR10-1*, *GuARR11* in roots and *GuARR1*, *GuARR2*, *GuARR10-2*, GuARR12 in aboveground part showing different responses in the qRT-PCR analysis, which should be further verified in the future.

**Figure 9:**
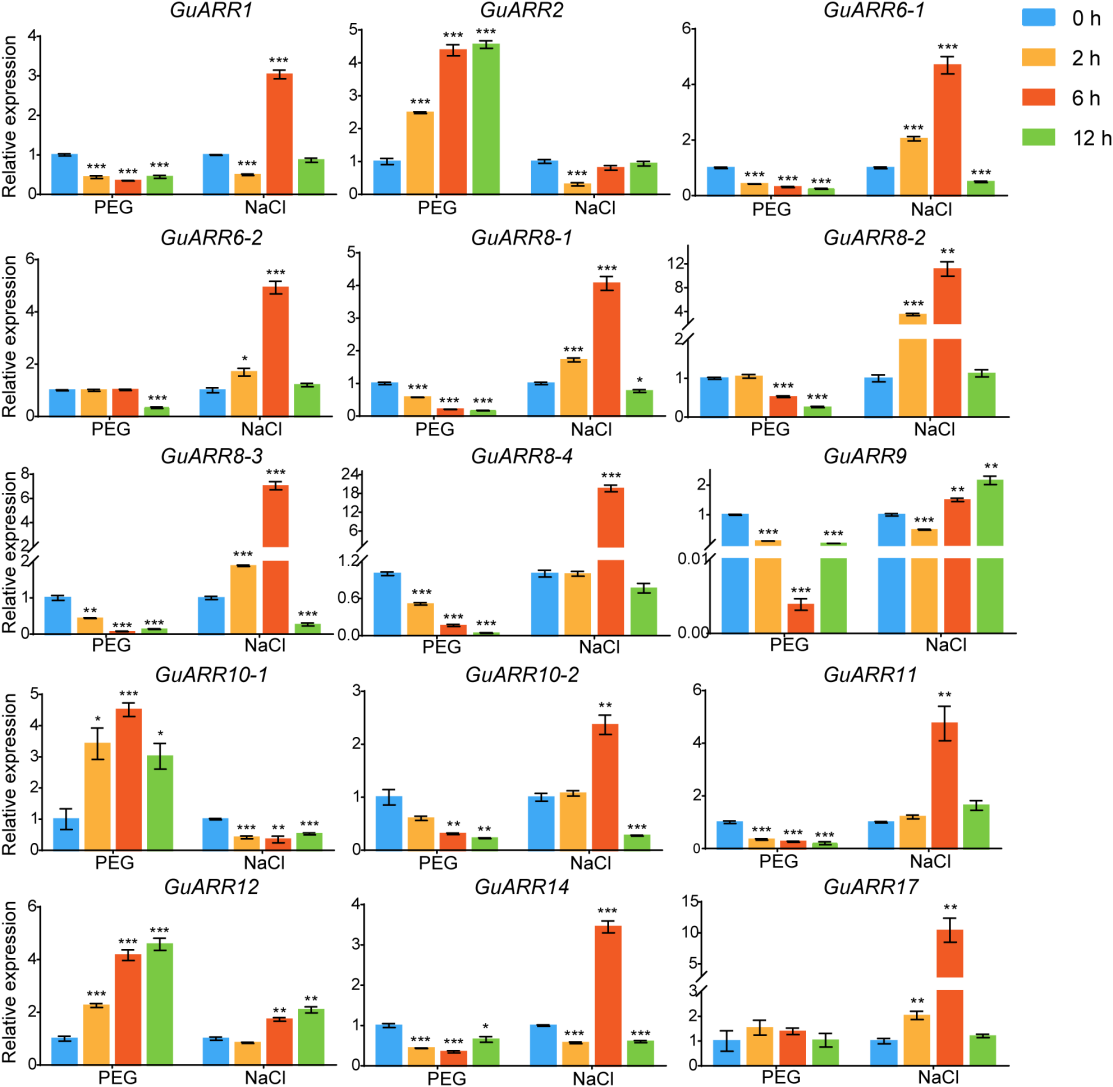
Expression profiles of *GuARRs* in response to PEG and NaCl treatments based on qRT-PCR analysis. Samples were collected at 0 h, 2 h, 6 h and 12 h after PEG (10%) and NaCl (150 mM) treatment. Student’s t-test was used to assess significant differences. Significance levels: *, p < 0.05; **, p < 0.01,***, p < 0.001.

Additionally, the expression levels of *GuARRs* were measured via qRT-PCR under treatments of PEG and NaCl (**Figure 9**). The results indicated that *GuARR2*, *GuARR10-1*, and *GuARR12* were induced under PEG stress, whereas the expressions of *GuARR1*, *GuARR6-1*, *GuARR8-1/2/3/4*, *GuARR9*, *GuARR10-2*, *GuARR11*, and *GuARR14* were decreased under PEG treatment. Under NaCl treatment, *GuARR6-1/2*, *GuARR8-1/2/3*, *GuARR10-2*, *GuARR17*, and *GuARR11* were up-regulated at 2 h and peaked at 6 h, then their expressions decreased at 12 h. *GuARR1*, *GuARR8-4*, and *GuARR14* had the highest expression levels after NaCl treatment for 6 h. *GuARR12* and *GuARR9* were up-regulated at 6 h and 12 h, respectively, but *GuARR10-1* was reduced by NaCl treatment.

**Figure 10:**
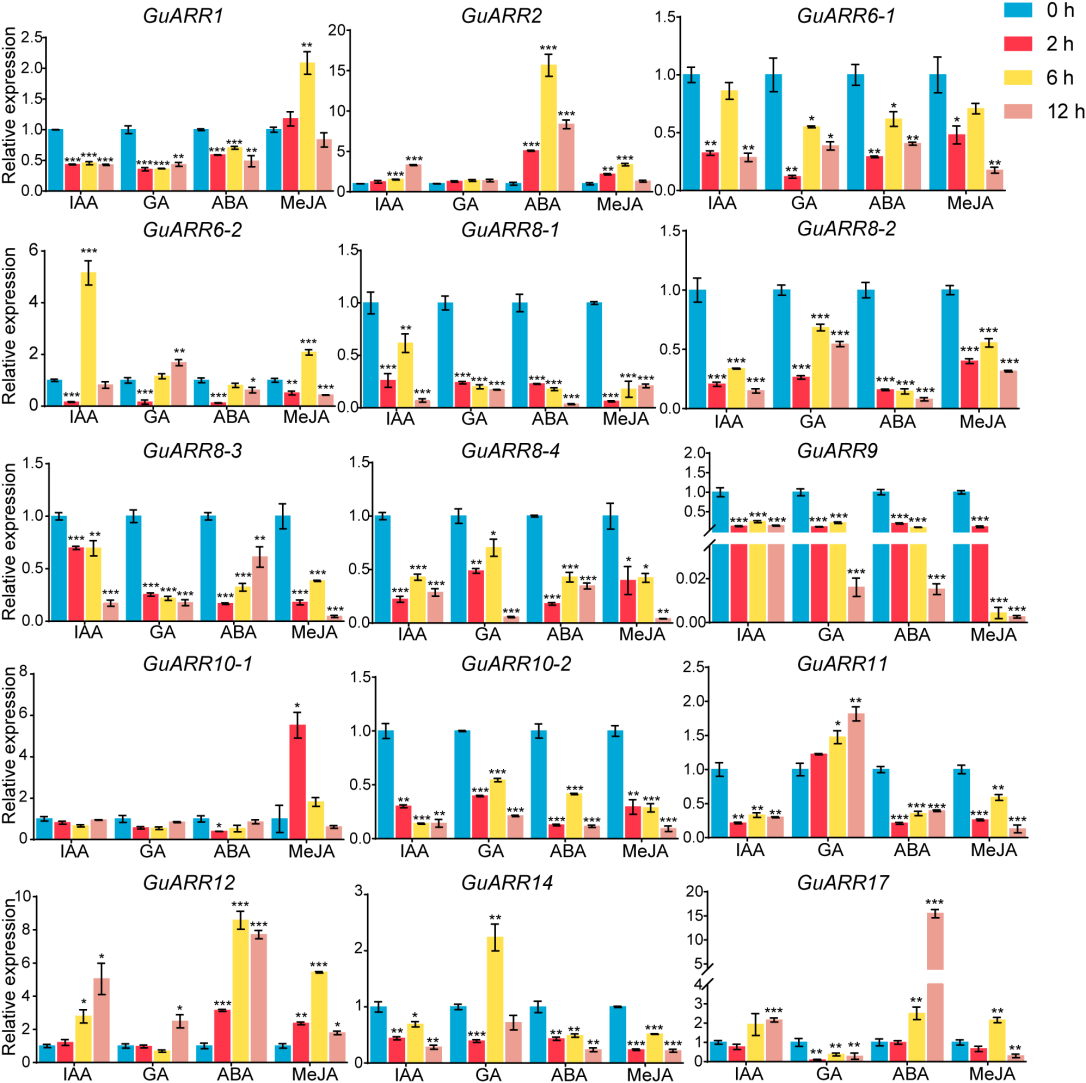
Expression profiles of *GuARRs* in response to IAA, GA, ABA and MeJA treatments based on qRT-PCR analysis. Samples were collected at 0 h, 2 h, 6 h and 12 h after the four kinds of hormone treatment. Student’s t-test was used to assess significant differences. Significance levels: *, p < 0.05; **, p < 0.01,***, p < 0.001.

Furthermore, the expression profiles of A-type and B-type *GuARRs* response to four kinds of phytohormones were explored using qRT-PCR (**Figure 10**). Under IAA treatment, *GuARR2*, *GuARR12* and *GuARR17* were induced at 6 h and 12 h, while *GuARR6-2* peaked at 6 h. Other *GuARRs*, including *GuARR1*, *GuARR6-1*, *GuARR8-1/2/3/4*, *GuARR9*, *GuARR10-2*, *GuARR11*, and *GuARR14*, were down-regulated after IAA treatment. The expression of *GuARR10-1* was not influenced obviously by IAA. Under GA treatment, *GuARR1*, *GuARR6-1*, *GuARR8-1/2/3/4*, *GuARR9*, *GuARR10-2*, and *GuARR17* were reduced, whereas *GuARR14* was induced significantly at 6 h. *GuARR12* and *GuARR6-2* were up-regulated at 12 h, and the expression of *GuARR11* gradually increased with the extension of treatment time. *GuARR2* and *GuARR10-1* did not displayed obvious change in their expressions. Most of the *GuARR* genes, including *GuARR1*, *GuARR6-1/2*, *GuARR8-1/2/3/4*, *GuARR9*, *GuARR10-1/2*, *GuARR11*, and *GuARR14*, were reduced by ABA treatment, but *GuARR2*, *GuARR12* and *GuARR17* were significantly induced. *GuARR12* was markedly up-regulated under MeJA treatment, peaking at 6 h. *GuARR1* and *GuARR2* were up-regulated at 2 h and continued to increase at 6 h, then decreased under the sustained MeJA treatment. *GuARR6-2* and *GuARR17* had the highest expression level after 6 h of MeJA treatment. *GuARR10-1* was dramatically induced under 2 h treatment of MeJA. Other *GuARRs*, including *GuARR6-1*, *GuARR8-1/2/3/4*, *GuARR9*, *GuARR10-2*, *GuARR11*, and *GuARR14*, were down-regulated under MeJA treatment.

### 3.8 The B-type GuARRs bind to the promoters of A-type *GuARRs*

**Figure 11:**
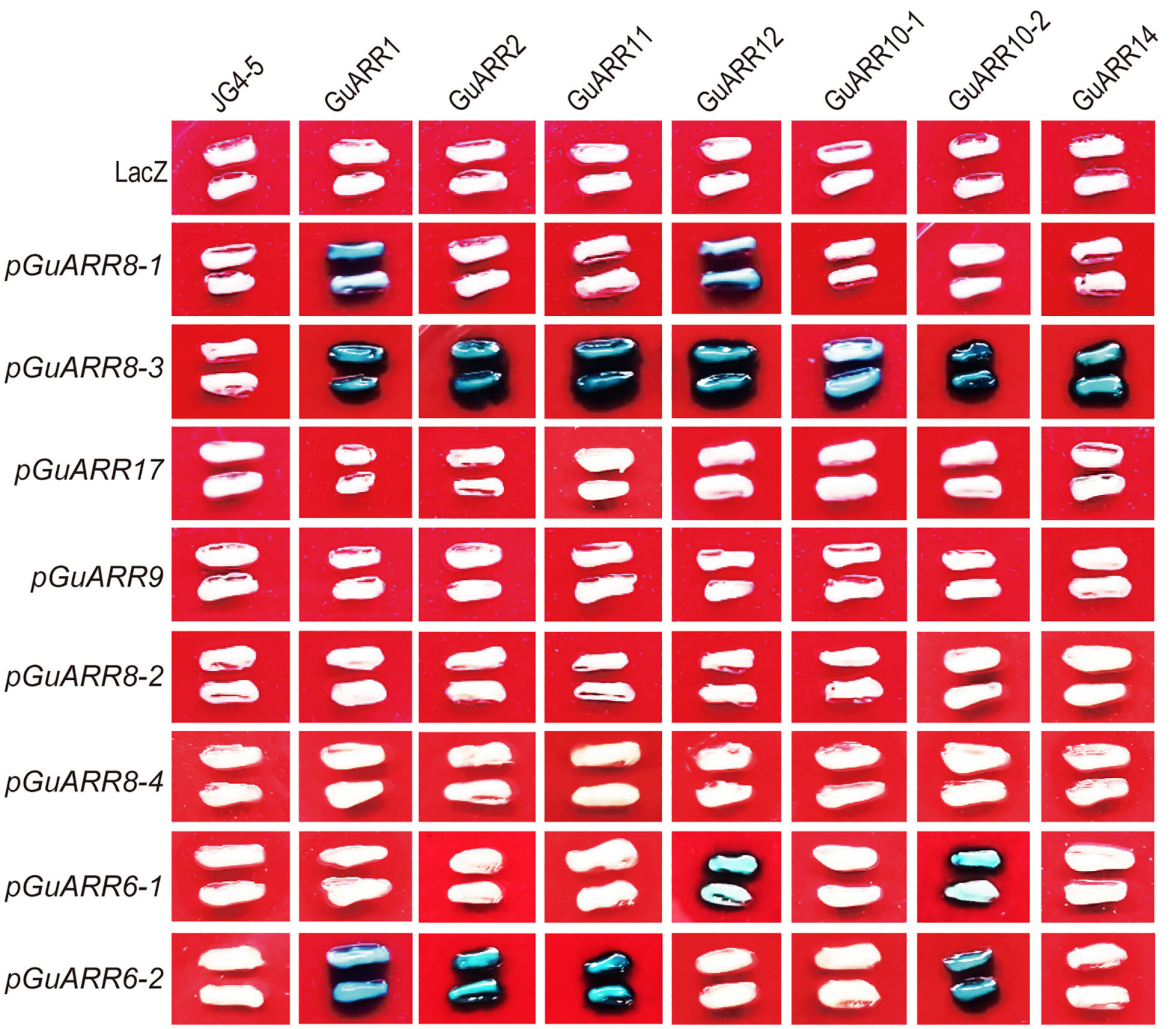
Systematic yeast one-hybrid assay showing the binding of B-type GuARRs to A-type *GuARRs* promoters. Blue yeast indicates the existence of the interaction.

To reveal the regulatory effect of B-type ARRs on A-type *ARR* genes, the binding between B-type licorice ARRs and A-type licorice *ARRs* promoters was examined using the yeast one-hybrid assay. The coding sequences of seven B-type *GuARRs*, namely *GuARR11*, *GuARR2*, *GuARR1*, *GuARR10-1*, *GuARR10-2*, *GuARR12* and *GuARR14* were inserted into the pJG45 vector as prey. Additionally, the promoters of eight *GuARRs* (*GuARR17*, *GuARR8-1/2/3/4*, *GuARR6-1/2*, *GuARR9*), whose expressions were induced under zeatin treatment, were cloned into the pLacZi vector as bait. And the lacZ reporter activity was firmly activated when *GuARR8-3* was concurrently transformed into the yeast cells with *GuARR1*, *GuARR2*, *GuARR11*, *GuARR12*, *GuARR10-1*, *GuARR10-2*, *GuARR14* and *GuARR8-1* with *GuARR1*, *GuARR12*. In addition, the lacZ reporter activity was activated when *GuARR6-1* was concurrently transformed with *GuARR12*, *GuARR10-2* as well as *GuARR6-2* with *GuARR1*, *GuARR2*, *GuARR11*, *GuARR10-2* (**Figure 11**). These findings provide evidence of the binding between B-type licorice ARRs and A-type licorice *ARRs* promoters, supporting the regulatory effect of B-type licorice ARRs on A-type licorice *ARRs*.

## 4 Discussion

Cytokinins are a kind of important plant hormones involved in the regulation of plant growth, development, and plant responses to stresses (Kieber and Schaller, 2018). Authentic response regulators (ARRs) are essential regulators of the widely used His-Asp phosphorelay system present in the cytokinin signaling (Hwang et al., 2012). With the development of bioinformatics, the ARR family gene members in some plants, including *Arabidopsis*, rice, tobacco, maize, and wild soybean have been studied (Chu et al., 2011; Hwang et al., 2012; Chen et al., 2018; Lv et al., 2021; Rehman et al., 2022). The B-type ARR families have also been analyzed in tomato, *B. napus*, and peach (Zeng et al., 2017; Wang et al., 2020; Jiang et al., 2022). However, the ARR family in licorice has not been examined. In this study, 17, 17, and 17 ARRs were identified and characterized from *G. glabra*, *G. uralensis* and *G. inflata* respectively, based on the presence of two conserved domains. The physicochemical properties, phylogenetic tree, chromosome locations, gene structures, structural domains, motif distribution, duplication events, *cis*-acting elements, and expression profiles after treatment with different hormones and abiotic stresses of these ARRs were analyzed, providing a basis for further research on *ARR* genes.

There were 35, 23, and 12 B-type ARRs identified in *B. napus*, peach, and tomato, respectively. *Arabidopsis*, rice, tobacco, maize, and wild soybean contained 21, 28, 59, 28, and 56 ARRs, respectively. The fewer ARRs in licorice than in other species may be a result of homologous gene loss during chromosome replication processes, which was similar in the *Jatropha curcas* (Geng et al., 2022). The length of amino acid sequences (124 to 700 aa), theoretical isoelectric point (4.89 to 8.69), and GRAVY varied significantly, suggesting the licorice ARRs were highly complex (**Table 1**, **Supplementary Table S3**), and comparable results were also obtained in some other species (Wang et al., 2020; Lv et al., 2021). The GRAVY of these ARRs was all negative (-0.916 to -0.042), indicating their hydrophily. Subcellular localization results exhibited that the licorice ARRs were all located in the nucleus, being similar as that in *B. napus* (Jiang et al., 2022), which may indicate that ARRs mainly function in nucleus and can interact with other transcription factors to modulate various biological processes. All 51 licorice ARRs had the conserved receiver domains harboring the D-D-K sites, which were also found in ARRs of other species (Lv et al., 2021; Geng et al., 2022). And the 21 B-type ARRs also contained a Myb-like DNA-binding domain, which plays a critical role in interacting with other regulators in cytokinin signaling pathways (Rubio et al., 2001; Safi et al., 2017). The locations of licorice *ARR* genes on chromosomes from *G. glabra* and *G. inflata* were similar, as they were all located on chr2, 3, 5, 6, 7, with chr2 and chr3 containing the most number of genes, and chr5 having the fewest number of 1 gene. Chr6 contained 4 genes, and chr7 contained 2 genes. Chr2 of *G. uralensis* also had the most number of 6 genes, and chr7 of *G. uralensis* had 2 genes (**Figure 4**). The results of chromosomal locations indicated the similarity of the 3 kinds of licorice species.

According to the phylogenetic tree, the 51 licorice ARRs were subdivided into three groups, with A-, B-, and C-type ARRs included in the three groups, respectively, which was consistent with the *Arabidopsis* ARRs (Kiba et al., 2004). Most of the A-type *ARRs* had 5 exons with 4 introns and the 3’ and 5’ UTR. Most B-type *ARRs* contained 6 exons with 5 introns and the 3’ and 5’ UTR, while the C-type *ARRs* contained only 2 exons without UTR regions (**Figure 3D**). The exons of B-type *ARRs* were longer than that of the other types of *ARRs*. Recently, introns have been proved to play an essential part in regulating gene expression (Monteuuis et al., 2019). The varied intron number among different types of *ARRs* might contribute to licorice *ARRs* evolution and function diversity, which were also found in other species (Jiang et al., 2022). Interestingly, *GgARR9-2* is much longer than other members in the entire family, reaching nearly 1.8 kb with a pretty long intron sequence, which might contribute to the biological function of this gene. A similar phenomenon was found in *NtARR27* (Lv et al., 2021). Moreover, the gene structure, conserved motifs numbers and distribution of these ARRs were similar in the same group but differed among different groups, thus licorice ARRs in the same group may possess similar biological functions. Gene duplication events act as a significant factor in expansions and realignments of genomes (Rehman et al., 2022). Plenty of transcription factor families have been reported to experience gene duplication events, like bHLH, NAC, and R2R3-MYB families (He et al., 2022; Liang et al., 2023; Yang et al., 2023). We found 3, 5, and 6 pairs of segmental duplication events in *GuARRs*, *GiARRs*, and *GgARRs*, respectively (**Figure 5**), while there were no tandem duplications identified, indicating that segmental duplications were the main driving force for the expansion of licorice *ARR* genes. Additionally, comparative syntenic maps among the three licorices were constructed. The results showed that 13 *GuARRs* had syntenic relationships with 15 *GgARRs*, 15 *GuARRs* and 15 *GiARRs* had syntenic relationships, while 15 *GgARRs* and 13 *GiARRs* had syntenic relationships (**Figure 6**). Most of these gene pairs bore a ratio of Ka/Ks below 1 (**Supplementary Table S4**), indicating that they underwent purifying selection in evolution with relatively conserved functions. The *cis*-acting elements in licorice *ARRs* promoter regions were analyzed and the presence of important *cis*-acting elements were found, showing that licorice *ARRs* may function in the regulation of plant growth, stress, and hormone responses (**Figure 7**; **Supplementary Table S5**). Except *GgARR3*, all the licorice A-type *ARRs* promoters contained the MYB or MYB recognition site *cis*-acting elements, indicating that these A-type licorice *ARRs* may interact with the B-type licorice *ARRs*. Promoters of three A-type *ARRs* (*GgARR6-2*, *GuARR6-2*, and *GiARR6-2*) contained a MYB binding site related to the process of flavonoid biosynthesis, which may bind to the Myb-like DNA-binding domain in B-type ARRs, thereby regulating flavonoid biosynthesis (Mason et al., 2005).

Strongly linked to their biological roles, the expression of genes exhibit diverse profiles under specific environmental conditions. Zeatin is a natural plant cytokinin present in higher plants. According to the RNA_seq data, most of the *GuARRs* exhibited similar expression patterns in the aboveground part and root under zeatin treatment, which may indicate that the *GuARRs* had similar regulatory mechanism in aboveground part and root. The expressions of *GuARR8-1/2/3*, *GuARR6-1/2*, *GuARR9* in the aboveground part and all the A-type *GuARRs* in the root were significantly induced by zeatin treatment, corresponding to their characristic of cytokinin response, thus indicating that A-type *GuARRs* may be involved in the cytokinin signaling (Kieber and Schaller, 2018). The transcription levels of A-type *GuARRs* under zeatin treatment explored by the qRT-PCR method generally coincided that in the RNA_seq data, while this was less so for the B-type *GuARRs*. This result was similar to the discovery of a past research in *Jatropha curcas* (Geng et al., 2022). This may be due to the fact that B-type *ARRs* expressions were not significantly influenced by cytokinin. Compared with B-type *ARRs*, A-type *ARRs* were more susceptible to induction by cytokinin in *Arabidopsis* and wheat (Gahlaut et al., 2014; Nguyen et al., 2016). The differences between the expression profiles of A- and B-type *GuARRs* might suggest the diverse functions of A- and B-type *GuARRs*.

Roots directly perceive the environmental stress underground. Therefore, root samples from licorice treated with PEG, NaCl, and various phytohormones were used for gene expression analyses response to abiotic stresses and phytohormones using the qRT-PCR method. Plenty of studies have shown that *ARR* genes are involved in responses to abiotic stresses. For example, ARR5 acts as a positive regulation factor in drought tolerance, and overexpression of *ARR5* in plants exhibited more tolerant to drought and hypersensitive to ABA (Huang et al., 2018). ARR1 and ARR12 are involved in promoting the sodium to accumulate in the shoots through modulating the transmittion of sodium ions to the shoot from roots in *Arabidopsis* (Mason et al., 2010). ARR1/10/12 negatively regulates plant drought stress response, and their triple mutants showed the phenotypes of more tolerance to drought than the wild type (Nguyen et al., 2016). In this study, *GuARR2*, *GuARR10-1*, and *GuARR12* were significantly induced by drought treatment. Thirteen out of 15 detected *GuARRs* showed increased trends under NaCl treatment, and 11 out of them, including *GuARR6-1/2*, *GuARR8-1/2/3/4*, *GuARR10-2*, *GuARR17*, *GuARR11*, *GuARR1*, and *GuARR14*, had the highest expression levels after 6 h of NaCl treatment, and then their expressions decreased at 12 h. *GuARR12* and *GuARR9* were up-regulated at 6 h and 12 h (**Figure 9**). This result indicated that these *GuARRs* can be induced by short-term NaCl stress at the proper concentration but reduced by continuous stress, which might be due to the long-term stress causing a certain degree of damage to plants. Overall, these results indicated that the *GuARR* genes play underlying roles in drought and NaCl stress responses. Phytohormones, being involved in various signal transduction, exert meaningful roles in plant growth and development. Previous reportes indicated that tomato B-type *SlARR* genes and *CcRR* genes exhibited different expressions patterns in response to different phytohormones (Wang et al., 2020; Tao et al., 2023). In addition, *BuARRs* in *B. napus* were found to respond to ABA treatment (Jiang et al., 2022). To better understand the roles of *GuARRs* in phytohormone response, the expression levels of *GuARRs* under four kinds of phytohormone treatments were measured using the qRT-PCR method, the results illustrated that *GuARRs* exhibited heterogeneous expression patterns under these phytohormone treatments. Interestingly, *GuARR2*, *GuARR12* and *GuARR17* showed up-regulated trends under IAA treatment. *GuARR11* and *GuARR12* showed increased trends under GA treatment (**Figure 10**). *GuARR2* and *GuARR12* were markedly induced by ABA and MeJA treatment and *GuARR1* was up-regulated after MeJA treatment for 6 h. It was reported that the *SlARR-B1/2/12* in tomato reached the highest expression after treating with MeJA for 6 h (Wang et al., 2020). *CcRR1/2/12a* were up-regulated upon MeJA treatment and *CcRR2* was induced by ABA (Tao et al., 2023). These analyses indicated the essential function of these *GuARRs* in licorice phytohormone responses. *GuARRR12* showed noticeable inductions under all four kinds of phytohormones, indicating a vital roles in the response to phytohormones.

The B-type ARRs possess a Myb-like DNA-binding domain besides the N-terminal receiver domain, which are transcriptional activators in the nucleus and can interact with a set of targets, including the A-type ARRs (Mason et al., 2005). In cytokinin signaling, A-type ARRs serve mainly as negative-feedback regulation factors (Kieber and Schaller, 2018). In this study, all of the A-type *GuARR* genes had the MYB or MYB recognition site *cis*-acting elements and were induced by zeatin. All eight A-type *GuARRs* and seven B-type *GuARRs* were chosen to examine the binding of B-type GuARRs to A-type *GuARRs* promoters using the yeast one-hybrid assay. The results indicated that GuARR1, GuARR2, GuARR11, GuARR12, GuARR10-1, GuARR10-2, GuARR14 can bind to the promoter of *GuARR8-3*, and GuARR1, GuARR12 can bind to the *GuARR8-1* promoter. GuARR1, GuARR2, GuARR11 and GuARR10-2 can bind to the *GuARR6-2* promoter and GuARR12 and GuARR10-2 can bind to the *GuARR6-1* promoter and may regulate their transcription levels (**Figure 11**). This study provide valuable information for further function research and complex regulatory mechanism of licorice *ARR* family genes and the further development of licorice functional genomics research.

## 5 Conclusions

In this study, a total of 51 ARRs were identified in *G. uralensis*, *G. inflata*, and *G. glabra* and were subclassified into three types, A-, B-, and C-type ARRs. A comprehensive analysis of these ARRs, including their evolutionary relationships, physicochemical characteristics, gene structures, chromosomal distributions and *cis*-acting elements was conducted. Notably, several stress and hormone response *cis*-elements were found in these licorice *ARR* genes promoters, showing their latent involvement in stress and hormone signaling pathways. Segmental duplication events were revealed to play a significant role in the licorice *ARR* genes expansion. These events likely favour to the diversity and complexity of the licorice ARR gene family. Furthermore, the temporospatial expression patterns of *GuARRs* response to abiotic stress and phytohormones were further characterized by the way of RNA-seq and qRT-PCR analysis, thus suggesting the wide involvement of *GuARRs* in responses to PEG, NaCl stress, and various phytohormones. Additionally, A- and B-type *GuARRs* function differently in cytokinin signaling, emphasizing the importance of understanding the distinct roles of different types of ARRs in plant development and stress responses. Lastly, the yeast one-hybrid assay provided evidence that GuARR1, GuARR2, GuARR11, GuARR12, GuARR10-1, GuARR10-2 and GuARR14 binds to the promoter of *GuARR8-3*, and GuARR1, GuARR12 are able to bind to the promoter of *GuARR8-1*. GuARR1, GuARR2, GuARR11 and GuARR10-2 can bind to the *GuARR6-2* promoter and GuARR12 and GuARR10-2 can bind to the *GuARR6-1* promoter. Overall, these findings shed an important light on the function and regulatory mechanisms of *ARR* genes in Chinese licorice. Further research is required to fully elucidate their functions and roles in plant growth, development, and stress responses. The insights gained from this study will be devoted to a deeper understanding of cytokinin signaling and its impact on plant physiology, which could have significant implications for agricultural and biotechnological applications.

## Data availability statement

The original contributions presented in the study are included in the article/**Supplementary Material**. Further inquiries can be directed to the corresponding authors.

## Author contributions

YS: Formal analysis, Investigation, Writing – original draft. GD: Formal analysis, Investigation, Writing – original draft, Writing – review & editing. HS: Formal analysis, Writing – review & editing. ZL: Writing – review & editing. HL: Writing – review & editing, Funding acquisition, Project administration, Supervision. GX: Funding acquisition, Project administration, Supervision, Writing – review & editing, Conceptualization.
